# Multicenter Comparison Study of both Analytical and Clinical Performance across Four Roche Hepatitis C Virus RNA Assays Utilizing Different Platforms

**DOI:** 10.1128/JCM.02193-16

**Published:** 2017-03-24

**Authors:** Johannes Vermehren, Evelyn Stelzl, Benjamin Maasoumy, Veronique Michel-Treil, Caterina Berkowski, Ed G. Marins, Ellen E. Paxinos, Enrique Marino, Heiner Wedemeyer, Christoph Sarrazin, Harald H. Kessler

**Affiliations:** a Medizinische Klinik 1, Universitätsklinikum Frankfurt, Frankfurt, Germany; b Institut für Hygiene, Mikrobiologie und Umweltmedizin, Medizinische Universität Graz, Graz, Austria; c Klinik für Gastroenterologie, Hepatologie und Endokrinologie, Medizinische Hochschule Hannover, Hannover, Germany; d Covance Central Laboratory Services, Geneva, Switzerland; e Roche Molecular Systems, Pleasanton, California, USA; University of Texas Medical Branch

**Keywords:** assay development, hepatitis C

## Abstract

The efficacy of antiviral treatment for chronic hepatitis C virus (HCV) infection is determined by measuring HCV RNA at specific time points throughout therapy using highly sensitive and accurate HCV RNA assays. This study compared the performances of two recently developed real-time PCR HCV RNA assays, cobas HCV for use on the cobas 6800/8800 systems (cobas 6800/8800 HCV) and cobas HCV for use on the cobas 4800 system (cobas 4800 HCV), with those of two established assays, the Cobas AmpliPrep/Cobas TaqMan HCV quantitative test, version 2 (CAP/CTM v2) and the Cobas TaqMan HCV test, version 2 for use with the High Pure system (HPS/CTM v2). The limits of detection (LODs) and linearity at lower concentrations (5 to 1000 IU/ml) were assessed for cobas 6800/8800 HCV and cobas 4800 HCV using WHO standard traceable panels representing HCV genotypes (GT) 1 to 4. Pairwise assay comparisons were also performed using 245 clinical samples representing HCV GT 1 to GT 4. Results from cobas 6800/8800 HCV and cobas 4800 HCV were linear at low HCV RNA concentrations (<0.3 log_10_ IU/ml difference between expected and observed results) with LODs of 8.2 IU/ml and 11.7 IU/ml, respectively, for GT 1. The new assays showed excellent agreement with results from CAP/CTM v2 and HPS/CTM v2 in samples with quantifiable viral loads. The concordances using the 6 million IU/ml cutoff were high among all four assays (90 to 94%). In conclusion, the cobas 6800/8800 HCV and cobas 4800 HCV tests are sensitive and linear and correlate well with the established Roche assays used in clinical practice.

## INTRODUCTION

Chronic hepatitis C virus (HCV) infection is a leading cause of chronic liver disease, cirrhosis, and hepatocellular carcinoma (http://www.who.int/mediacentre/factsheets/fs164/en/). The curing of HCV infection is associated with reduced liver-related morbidity and mortality and an improved quality of life (1). Following the introduction of direct-acting antivirals (DAAs), HCV infection can now be cured in the majority of patients (2–7).

Treatment efficacy is determined by measuring HCV RNA at specific time points before, during, and after antiviral therapy. According to current guidelines, real-time PCR-based HCV RNA assays must be highly sensitive and accurate, with a broad linear range of quantification across all HCV genotypes (8, 9).

Baseline viral load is known to be an important predictor of treatment efficacy in patients treated with interferon (IFN)-based regimens (10). A low baseline HCV RNA, along with other factors, has also been used to identify patients eligible for a shortened course of IFN-based therapy (11, 12). Moreover, on-treatment HCV RNA measurements were used to guide treatment duration and decisions on futility, particularly in patients treated with the protease inhibitors telaprevir and boceprevir (13, 14). Even with today's widely used IFN-free ledipasvir/sofosbuvir regimen, baseline HCV RNA is used for determining treatment duration (4), whereas subsequent HCV RNA measurements are mainly used for assessing the patient's adherence and for demonstrating viral eradication after treatment completion. In addition, on-treatment HCV RNA measurements may still be used for predicting the efficacy of IFN-free DAA regimens in some patient subgroups, including in patients with cirrhosis and those with HCV genotype (GT) 3 infections (15, 16). For this purpose, current guidelines recommend measuring HCV RNA at different time points, ideally with the same assay to maintain consistency of results (8, 9).

Real-time PCR-based assays are currently the most widely used methods for quantifying HCV RNA (17, 18). These include the Cobas TaqMan HCV test, version 2 for use with the High Pure system (HPS/CTM v2) and the Cobas AmpliPrep/Cobas TaqMan HCV quantitative test, version 2 (CAP/CTM v2) utilizing a dual-probe design for improved genotype inclusivity (17).

More recently, two new cobas HCV real-time PCR-based assays were developed, namely the cobas HCV test for use on the cobas 6800 and cobas 8800 systems (cobas 6800/8800 HCV) and the cobas HCV test for use on the cobas 4800 system (cobas 4800 HCV). For both assays, the same dual-probe design known from the CAP/CTM v2 test was adopted. The cobas 6800/8800 HCV test has been *in vitro* diagnostics (IVD)/Conformité Européenne (CE)-labeled and U.S. FDA approved. The cobas 4800 HCV test has the IVD/CE mark and it is not available in the United States. Despite this, real-world studies are required for establishing the clinical performance of the new assays to ensure consistency of results. The aim of this study was to determine the performance characteristics of the cobas 6800/8800 HCV and cobas 4800 HCV tests using clinical samples representing all major HCV genotypes and to compare them with those of two established HCV RNA assays, HPS/CTM v2 and CAP/CTM v2.

## RESULTS

### Analytical performance.

Values for the accuracy, precision, and sensitivity (limit of detection [LOD]) were calculated for the cobas 6800/8800 HCV and cobas 4800 HCV tests for each genotype (see Table S1, S2, S3, and S4 in the supplemental material). For cobas 6800/8800 HCV, cobas 4800 HCV, CAP/CTM v2, and HPS/CTM v2, the LODs for HCV GT 1 were 8.2 IU/ml (95% confidence interval [CI], 6.7 to 14.4), 11.7 IU/ml (95% CI, 8.9 to 21.5), 14.4 IU/ml (95% CI, 12.0 to 19.0), and 5.22 IU/ml (95% CI was not calculable as the fiducial limits of the probit curve could not be calculated at the 95% confidence level with the data pattern observed), respectively. The probit curves fit to the observed hit rates of the panel members for cobas 6800/8800 HCV and cobas 4800 HCV can be seen in Fig. S1 and Fig. S2, respectively, and the curves for CAP/CTM v2 and HPS/CTM v2 can be seen in Fig. S3 and Fig. S4, respectively, in the supplemental material.

For cobas 6800/8800 HCV, cobas 4800 HCV, and CAP/CTM v2, the observed LODs for HCV GT 2, GT 3, and GT 4 were below 15 IU/ml for each of the genotypes, except for HCV GT 4 run on cobas 4800 HCV and on CAP/CTM v2. While the LOD for cobas 6800/8800 HCV for GT 4 was 13.7 IU/ml (95% CI, 10.5 to 21.6), it exceeded the LOD of 15 IU/ml for cobas 4800 HCV (18.4 IU/ml [95% CI, 14.2 to 27.6 IU/ml]) and CAP/CTM v2 (19.5 IU/ml [95% CI, 14.5 to 32.6]) (Tables S1, S3, and S4).

The observed mean log_10_ IU/ml titers from each of the 5 panel members (1000, 100, 50, 25, and 15 IU/ml) were plotted against the nominal HCV RNA log_10_ IU/ml concentrations. Individual accuracy values (mean log_10_ observed − log_10_ nominal) ranged from 0 to 0.34 log_10_ IU/ml for cobas 6800/8800 HCV, and from −0.23 to 0.29 log_10_ IU/ml for cobas 4800 HCV. Linearity (observed mean [log_10_ IU/ml] − linearized [log_10_ IU/ml]) was within 0.15 and 0.16 log_10_ IU/ml of nominal for cobas 6800/8800 HCV and cobas 4800 HCV, respectively (Table S2).

### Clinical performance.

**FIG 1:**
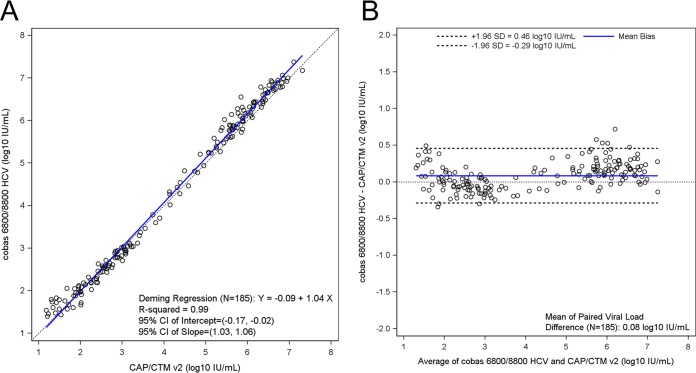
(a) Deming regression plot for cobas 6800/8800 HCV versus CAP/CTM v2 (*n* = 185). (b) Bland-Altman plot for cobas 6800/8800 HCV versus CAP/CTM v2 (*n* = 185). CAP/CTM v2, Cobas AmpliPrep/Cobas TaqMan HCV quantitative test; CI, confidence interval.

**FIG 2:**
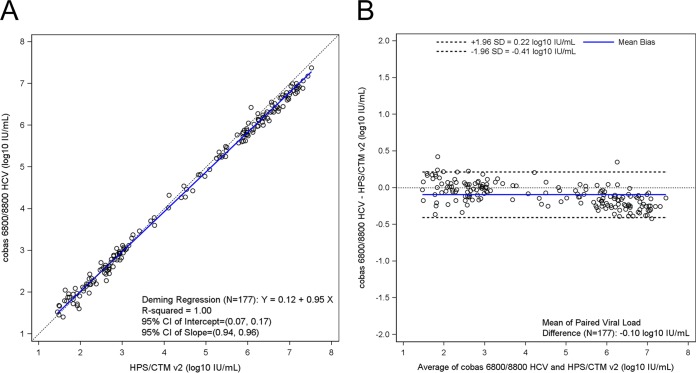
(a) Deming regression plot for cobas 6800/8800 HCV versus HPS/CTM v2 (*n* = 177). (b) Bland-Altman plot for cobas 6800/8800 HCV versus HPS/CTM v2 (*n* = 177). CI, confidence interval; HPS/CTM v2, cobas TaqMan HCV Test, version 2 for use with the High Pure system.

**FIG 3:**
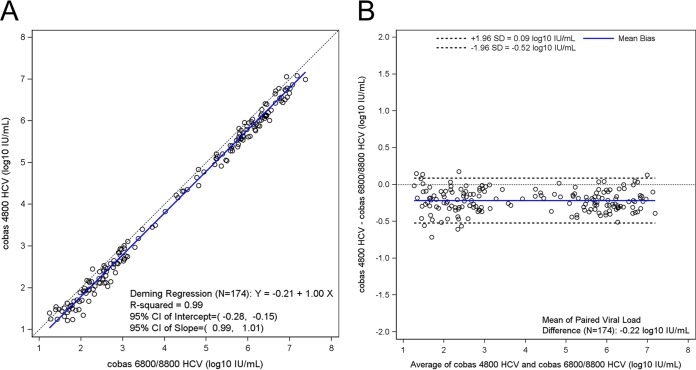
(a) Deming regression plot for cobas 6800/8800 HCV versus cobas 4800 HCV (*n* = 174). (b) Bland-Altman plot for cobas 6800/8800 HCV versus cobas 4800 HCV (*n* = 174).

Pairwise method comparisons across all four assays are summarized in Table 1. A total of 245 clinical samples were tested with all 4 assays, and the results within the linear ranges were used in pairwise method comparisons (see Table S5, S6, S7, S8, S9, and S10). The mean differences for any given pairwise comparison did not exceed 0.31 log_10_ IU/ml and ranged from 0.08 log_10_ IU/ml between cobas 6800/8800 HCV and CAP/CTM v2 to 0.31 log_10_ IU/ml between cobas 4800 HCV and HPS/CTM v2 (Fig. 1, 2, and 3; see also Fig. S5, S6, and S7).

**TABLE 1:** Pairwise method comparisons across four Roche platforms using clinical HCV specimens

Comparison^*a*^	No. of paired tests^*b*^	Slope	Intercept	*R*^2^	Mean difference (log_10_ IU/ml [95% CI])
cobas 6800/8800 HCV vs CAP/CTM v2	185	1.04	−0.09	0.992	0.08 (0.06 to 0.11)
cobas 6800/8800 HCV vs HPS/CTM v2	177	0.95	0.12	0.996	−0.10 (−0.12 to −0.07)
cobas 4800 HCV vs cobas 6800/8800 HCV	174	1.00	−0.21	0.994	−0.22 (−0.24 to −0.20)
cobas 4800 HCV vs CAP/CTM v2	170	1.05	−0.33	0.988	−0.14 (−0.17 to −0.10)
cobas 4800 HCV vs HPS/CTM v2	162	0.95	−0.10	0.992	−0.31 (−0.34 to −0.28)
HPS/CTM v2 vs CAP/CTM v2	172	1.11	−0.31	0.991	0.17 (0.13 to 0.21)

a CAP/CTM v2, Cobas AmpliPrep/Cobas TaqMan HCV quantitative test, version 2; HCV, hepatitis C virus; HPS/CTM v2, Cobas TaqMan HCV test, version 2 for use with the High Pure system.

b Only valid paired results within the respective common linear ranges were included in the calculations.

### Comparison of cobas 6800/8800 HCV and CAP/CTM v2.

Of the 245 samples tested by cobas 6800/8800 HCV and CAP/CTM v2, 196 had detectable viral loads on both platforms, with an overall concordance of 98%, and 185 samples gave results within the linear ranges of both assays (Table S5). Moreover, 103/110 (94%) samples were concordant with respect to their classifications using the 6 million IU/ml cutoff (i.e., viral loads either less than or greater than 6 million IU/ml). Each of the discordant samples had viral loads greater than 6 million IU/ml by cobas 6800/8800 HCV and less than 6 million IU/ml by the CAP/CTM v2 (GT 1a, *n* = 1; GT 1b, *n* = 6). On the basis of a Deming regression analysis, the coefficient of regression (*R*^2^) among the detected samples within the linear range was 0.99 (Fig. 1a). The mean difference in quantifiable samples was 0.08 log_10_ IU/ml HCV RNA (95% CI, 0.06 to 0.11) between the two assays, indicating that cobas 6800/8800 HCV returned slightly higher titers than CAP/CTM v2 (Fig. 1b).

### Comparison of cobas 6800/8800 HCV and HPS/CTM v2.

Of the 245 samples tested by cobas 6800/8800 HCV and HPS/CTM v2, 196 had detectable viral loads on both platforms, with an overall concordance of 98%, and 177 gave results within the linear ranges of both assays. One hundred of 109 (92%) samples were concordant with respect to their classifications using the 6 million IU/ml cutoff. Each of the discordant samples had viral loads less than 6 million IU/ml according to cobas 6800/8800 HCV and greater than 6 million IU/ml with HPS/CTM v2 (GT 1a, *n* = 5; GT 1b, *n* = 3; GT 1 unclassified, *n* = 1). On the basis of a Deming regression analysis, the coefficient of regression among the detected samples within the overlapping linear range was 0.996 (Fig. 2a). The mean difference in quantifiable samples was −0.10 log_10_ IU/ml HCV RNA (95% CI, −0.12 to −0.07) between the two assays, indicating that cobas 6800/8800 HCV returned slightly lower titers than HPS/CTM v2 (Fig. 2b).

### Comparison of cobas 6800/8800 HCV and cobas 4800 HCV.

Of the 245 samples tested by cobas 6800/8800 HCV and cobas 4800 HCV, 182 showed quantifiable viral loads on both assays, with an overall concordance of 97%, and 174 samples were within the linear ranges of both assays. Eighty-five of 94 (90%) samples were concordant with regard to the 6 million IU/ml cutoff. Each of the discordant samples had viral loads greater than 6 million IU/ml with cobas 6800/8800 HCV and lower than 6 million IU/ml according to cobas 4800 HCV. On the basis of Deming regression analysis, the coefficient of regression among detected samples within the overlapping linear range was 0.99 (Fig. 3a). The mean difference in quantifiable samples was −0.22 log_10_ IU/ml HCV RNA (95% CI, −0.24 to −0.20) between the two assays, indicating that cobas 6800/8800 HCV returned higher HCV RNA titers than cobas 4800 HCV (Fig. 3b).

Results from Deming regression analyses and Bland-Altman plots of other method comparisons are shown in Fig. S5, S6, and S7 in the supplemental material.

## DISCUSSION

In this study, the cobas 6800/8800 HCV and cobas 4800 HCV tests had detection levels that meet the requirements of current HCV treatment guidelines that recommend using a highly sensitive real-time PCR assay with an LOD ≤15 IU/ml (EASL) or ≤25 IU/ml (AASLD) for viral load monitoring (8, 9). In HCV GT 4 panels, the LOD was estimated to be less than 15 IU/ml for cobas 6800/8800 HCV, but was found to be slightly above this cutoff for cobas 4800 HCV. The clinical relevance of these findings could not be explored due to a lack of GT 4 samples in our clinical set (only 1 sample was known to be GT 4). This supports the need for assay standards representing other HCV genotypes, which are known to be genetically highly variable. The WHO HCV international standard comprises only subtype a of GT 1 (HCV GT 1a) plasma (19).

The cobas 6800/8800 HCV and cobas 4800 HCV tests were accurate even at the lower end of the linear range (15 to 1000 IU/ml). As with the second version of the CAP/CTM v2 test, both assays provide a single value for the LOD and the lower limit of quantification (LLOQ). Although on-treatment HCV RNA measurement may play only a small role for outcome prediction in the era of DAAs (16), highly sensitive and accurate HCV RNA quantification is still required during and after antiviral therapy to document treatment adherence and successful HCV eradication (20, 21).

The second version of the CAP/CTM test was developed to provide improved sensitivity and accuracy with a smaller sample input volume (17). The CAP/CTM v2 also contains a dual-probe design to improve genotype inclusivity, because the previous version 1 of the CAP/CTM did not always correctly quantify some genotypes and subtypes (22, 23). This dual-probe design was also incorporated into the cobas 6800/8800 HCV and cobas 4800 HCV tests, which were developed to meet the requirements of medium-to-high and low-to-medium throughput laboratory facilities and to have a minimal hands-on time.

In this study, the concordance between cobas 6800/8800 HCV and cobas 4800 HCV was very high, exceeding 97% for determining results as “detectable” or “undetectable” in a large number of clinical samples (including samples with very low HCV RNA levels) representing HCV GT 1, which is the most common genotype in Europe and in the United States (24). Moreover, the results from cobas 6800/8800 HCV and cobas 4800 HCV were very highly correlated with those from the HPS/CTM v2 and the CAP/CTM v2 for samples with quantifiable viral loads.

Ledipasvir/sofosbuvir, currently one of the most widely used DAA regimens for treating HCV GT 1 infection, can be administered for a shortened 8-week duration in treatment-naive, noncirrhotic patients with baseline viral loads below 6 million IU/ml (http://www.accessdata.fda.gov/drugsatfda_docs/nda/2014/205834Orig1s000MedR.pdf). This recommendation is based on data from the ION-3 approval study, which used HPS/CTM v2 for viral load determination (4). This 6 million IU/ml cutoff is being applied in many countries, mainly for its cost-saving potential, despite the fact that its scientific basis has been questioned (25). This has sparked interest regarding the comparability of HPS/CTM v2 with other widely used assays. Particularly, it was shown that the rates of patients with HCV RNA levels higher than the 6 million cutoff may vary significantly with the test method used (26). For example, the number of patients with an HCV RNA >6 million IU/ml as measured by the Abbott RealTime HCV assay is significantly smaller than that measured by the CAP/CTM v2 test. Therefore, patients may be at risk for undertreatment depending on which assay is used to determine HCV RNA. Moreover, considerable HCV RNA variability can be expected for results from the same test measured at different time points.

In this study, cobas 6800/8800 HCV showed high levels of concordance with CAP/CTM v2 and HPS/CTM v2 with respect to the 6 million IU/ml cutoff. Similar concordances were observed between cobas 4800 HCV and the two established assays (data not shown). Our data show that the vast majority of HCV RNA concentrations measured with cobas 6800/8800 HCV and cobas 4800 HCV would be slightly higher than those measured by CAP/CTM v2 and slightly lower than those measured with HPS/CTM v2 at this clinical decision point.

The limitations of our study are that all three study sites were located in central Europe (Switzerland, Germany, and Austria) and each platform was represented by a single site. Moreover, archived samples used for testing did not comprehensively include all genotypes and subtypes. Consequently, less-common genotypes (i.e., GT 4 to GT 6) were not well represented. In addition, the genotype assignments were based on previous (historical) testing, and sample volumes limited confirmatory genotype testing.

In conclusion, our data show that the cobas 6800/8800 HCV and cobas 4800 HCV tests are highly sensitive and accurate. They are useful for quantifying baseline HCV RNA to determine treatment duration, to monitor patients during DAA therapy, and to demonstrate viral eradication. They correlate well with established Roche assays used in clinical practice. Further studies correlating these results and other system features (e.g., turn-around-time, throughput, workflow, and automation) to those of other platforms are warranted.

## MATERIALS AND METHODS

### Study sites and molecular assays.

This was an international multicenter clinical study, including sites located in Frankfurt, Germany (Medizinische Klinik 1, University Hospital Frankfurt), Geneva, Switzerland (Covance Laboratories), and Graz, Austria (Molecular Diagnostics Laboratory, Medical University of Graz). All experiments were performed in International Standard Organization (ISO) 9001:2008-certified or ISO 17,025:2005-accredited laboratories.

cobas 6800/8800 HCV and cobas 4800 HCV are quantitative nucleic acid tests that enable the detection and quantification of HCV RNA in EDTA plasma or serum from HCV-infected patients. Dual probes are used to detect and quantify, but not discriminate, HCV GT 1 to GT 6. HCV viral load is quantified against a non-HCV (noncompetitive) armored RNA quantification standard (RNA QS), which is introduced to each specimen at the beginning of sample preparation. The RNA QS also functions as an internal control for monitoring of the entire sample preparation and PCR amplification process. In addition to the RNA QS, three external controls are included in each run: the high-positive control, the low-positive control, and the HCV negative control.

Assays (cobas 6800/8800 HCV, cobas 4800 HCV, CAP/CTM v2, and HPS/CTM v2) were performed according to the manufacturer's instructions. All assays have been calibrated to the WHO international standard for HCV RNA, and results are reported in IU/ml.

### Analytical performance.

The analytical performance, including accuracy, precision, sensitivity, and linearity, was determined for the cobas 6800/8800 HCV and the cobas 4800 HCV assays by testing individual clinical HCV specimens representing HCV genotypes 1 through 4 as serial dilutions in HCV-negative (for HCV antibody and HCV RNA) single-donor plasma matrices. Briefly, for each genotype, a panel consisting of 7 members containing different HCV RNA concentrations (1000 IU/ml, 100 IU/ml, 50 IU/ml, 25 IU/ml, 15 IU/ml, 10 IU/ml, and 5 IU/ml) was tested with 40 replicates per member. For GT 1, the concentration of the parental stock was calibrated using a secondary standard traceable to the WHO international standard (19). As there are no recognized international standards for GT 2, GT 3, or GT 4, panels were built based on CAP/CTM v2 titers of parental stocks, and then reassigned using the Abbott m2000 system. The Abbott RealTime assay, a commonly used FDA-approved competitor assay, was used to minimize perceived or potential bias in assigning titers for the panel.

The linearity results were reported as average log_10_ (IU/ml) differences from the respective linearized values.

Accuracy and precision were assessed for both assays within the claimed linear range of each assay (Table 2). The accuracy was calculated as the log_10_ IU/ml difference between the observed mean and nominal concentrations. Precision was estimated by calculating a coefficient of variation for replicate measurements. The LOD was estimated by probit analysis, and the associated 95% CIs were calculated.

**TABLE 2:** Lower and upper limits of quantification and sample input and processing volumes for the 4 assays

Assay^*a*^	PCR target (probe type)^*b*^	Internal control^*c*^ (type)	Sample processing vol (ml)	Linear range^*d*^	
				LLOQ	ULOQ
HPS/CTM v2	5′ UTR (TaqMan)	QS (competitive armored RNA)	500	25	3.9 × 10^8^
CAP/CTM v2	5′ UTR (TaqMan dual probe)	QS (competitive armored RNA)	500	15	1.0 × 10^8^
cobas 4800 HCV	5′ UTR (TaqMan dual probe)	QS (noncompetitive armored RNA)	500	15	1.0 × 10^8^
cobas 6800/8800 HCV	5′ UTR (TaqMan dual probe)	QS (noncompetitive armored RNA)	500	15	1.0 × 10^8^

a CAP/CTM v2, Cobas AmpliPrep/Cobas TaqMan HCV quantitative test, version 2; HCV, hepatitis C virus; HPS/CTM v2, Cobas TaqMan HCV test, version 2 for use with the High Pure system.

b UTR, untranslated region.

c QS, quantification standard.

d LLOQ, lower limit of quantification; ULOQ, upper limit of quantification.

### Clinical performance.

For pairwise assay comparisons, Deming regression analyses were performed and Bland-Altman plots were constructed. A total of 245 clinical specimens left over from previous routine testing were tested by cobas 6800/8800 HCV and cobas 4800 HCV and compared to the results from testing with CAP/CTM v2 and HPS/CTM v2. We chose specimens spanning the linear ranges of each of the four assays, as well as samples with low HCV RNA titers and those with undetectable or detectable/< LLOQ results. Genotypes had been assigned previously using the Versant HCV genotype 2.0 assay (Siemens Healthcare, Erlangen, Germany). The samples comprised GT 1a (*n* = 137), GT 1b (*n* = 43), GT 1 of unknown subtype (*n* = 7), GT 2 (*n* = 11), GT 3 (*n* = 22), and GT 4 (*n* = 1). Twenty-four samples did not have assigned genotypes. Assay concordance was assessed in samples with viral loads >1 million IU/ml with respect to the clinically relevant 6 million IU/ml cutoff, which is being used to tailor treatment durations in patients receiving ledipasvir/sofosbuvir.

## Supplementary Material

Supplemental material
