# Supplementary material for: Multicenter Comparison Study of both Analytical and Clinical Performance across Four Roche Hepatitis C Virus RNA Assays Utilizing Different Platforms
Source: J Clin Microbiol. 2017 Mar 24;55(4):1131–9. doi: 10.1128/JCM.02193-16 (PMC5377840; doi:10.1128/JCM.02193-16)
Supplement: Supplemental material [file supp_55_4_1131__index.html]

Supplemental material 

# Multicenter Comparison Study of both Analytical and Clinical Performance across Four Roche Hepatitis C Virus RNA Assays Utilizing Different Platforms

## Supplemental material

- Supplemental file 1 -

  Tables S1 (Limits of detection for HCV Genotypes 1, 2, 3, and 4 by cobas 6800/8800 HCV and cobas 4800 HCV), S2 (Observed HCV RNA concentrations for genotypes 1 to 4 by cobas 6800/8800 HCV and cobas 4800 HCV), S3 (Limits of detection and observed HCV RNA concentrations for genotypes 1, 2, 3, and 4 by CAP/CTM v.2), S4 (Limits of detection and observed HCV RNA concentrations for genotype 1 by HPS/CTM v2), S5 (Distribution of sample results across cobas 6800/8800 HCV versus CAP/CTM HCV v2), S6 (Distribution of sample results across cobas 6800/8800 HCV versus HPS/CTM v2), S7 (Distribution of sample results across cobas 6800/8800 HCV versus cobas 4800 HCV), S8 (Distribution of sample results across cobas 4800 HCV versus CAP/CTM v2), S9 (Distribution of sample results across cobas 4800 HCV versus HCV HPS v2), and S10 (Distribution of sample results across HPS/CTM v2 versus CAP/CTM v2) and Fig. S1 (Limit of detection for cobas 6800/8800 HCV for genotype 1), S2 (Limit of detection for cobas 4800 HCV for genotype 1), S3 (Limit of detection estimation for CAP/CTM v2 for genotype 1), S4 (Limit of detection estimation for HPS/CTM v2 for genotype 1), S5 (Deming regression plot and Bland-Altman plot for cobas 4800 HCV versus CAP/CTM v2), S6 (Deming regression plot and Bland-Altman plot for cobas 4800 HCV versus HPS/CTM v2), and S7 (Deming regression plot and Bland-Altman plot for HPS/CTM v2 versus CAP/CTM v2)

  PDF, 1.1M
