# Supplementary material for: Multicenter Comparison Study of both Analytical and Clinical Performance across Four Roche Hepatitis C Virus RNA Assays Utilizing Different Platforms
Source: J Clin Microbiol. 2017 Mar 24;55(4):1131–9. doi: 10.1128/JCM.02193-16 (PMC5377840; doi:10.1128/JCM.02193-16)
Supplement: Supplemental material [file JCM.02193-16_zjm999095426s1.pdf]

Table S1: Limit of Detection (LoD) for HCV Genotypes 1, 2, 3, and 4 by cobas 6800/8800 HCV and cobas 4800 HCV

	Nominal Titer (IU/mL)[a]	Nominal Titer (log_{10} IU/mL)	Detection of HCV: cobas 6800/8800 HCV % (#)	Detection of HCV: cobas 4800 HCV % (#)
Genotype 1	1000	3.00	100 (40)	100 (40)
	100	2.00	100 (40)	100 (40)
	50	1.70	100 (40)	100 (40)
	25	1.40	100 (40)	100 (40)
	15	1.18	100 (40)	97.5 (39)
	10	1.00	97.5 (39)	90 (36)
	5	0.70	75 (30)	77.5 (31)
	**LoD by Probit [95% CI]**		**8.2 [6.7, 14.4]**	**11.7 [8.9, 21.5]**
Genotype 2	737	2.87	100 (40)	100 (40)
	74	1.87	100 (40)	100 (40)
	37	1.57	100 (40)	100 (40)
	18	1.26	100 (40)	97.5 (39)
	11	1.05	100 (40)	92.5 (37)
	7	0.87	95 (38)	87.5 (35)
	4	0.57	82.5 (33)	57.5 (23)
	**LoD by Probit [95% CI]**		**6.3 [4.9, 12.0]**	**12.5 [9.6, 20.5]**
Genotype 3	585	2.77	100 (40)	100 (40)
	59	1.76	100 (40)	100 (40)
	29	1.46	100 (40)	100 (40)
	15	1.16	100 (40)	100 (40)
	9	0.94	100 (40)	100 (40)
	6	0.77	90 (36)	90 (36)
	3	0.46	80 (32)	80 (32)
	**LoD by Probit [95% CI]**		**6.1 [4.6, 11.1]**	**6.1 [4.6, 11.1]**
Genotype 4	558	2.75	100 (40)	100 (40)
	56	1.75	100 (40)	100 (40)
	28	1.45	100 (40)	97.5 (39)
	14	1.14	94.9 (37/39)[b]	90 (36)
	8	0.92	87.5 (35)	75 (30)
	6	0.75	64.1 (25/39)[b]	57.5 (23)
	3	0.45	42.5 (17)	20.0 (8)
	**LoD by Probit [95% CI]**		**13.7 [10.5, 21.6]**	**18.4 [14.2, 27.6]**

[a] for GT2, 3, and 4, the values for the expected concentrations of each panel member were assigned using a linearization method on the mean observed titers from Abbott

[b] Only 39 replicates for Genotype 4, at both 1.14 and 0.75 log10 IU

Table S2: Observed HCV RNA concentration for genotypes 1-4 by cobas 6800/8800 HCV and cobas 4800 HCV

			Observed HCV RNA Concentration ($\log_{10}$ IU/mL)[a]							
			cobas 6800/8800 HCV				cobas 4800 HCV			
	Nominal Titer (IU/mL)[b]	Nominal Titer ($\log_{10}$ IU/mL)	Mean	SD [%CV]	Accuracy[c]	Linearity[d]	Mean	SD [%CV]	Accuracy[c]	Linearity[d]
Genotype 1	1000	3.00	3.04	0.07 [2.17]	0.04	-0.15	2.92	0.08 [2.79]	-0.08	-0.11
	100	2.00	2.15	0.08 [3.84]	0.15	-0.04	2.01	0.10 [5.03]	0.01	-0.02
	50	1.70	1.92	0.11 [5.64]	0.22	0.03	1.67	0.18 [10.52]	-0.03	-0.06
	25	1.40	1.65	0.18 [10.80]	0.26	0.07	1.46	0.14 [9.72]	0.06	0.03
	15	1.18	1.46	0.15 [10.06]	0.29	0.1	1.37	0.11 [8.13]	0.19	0.16
	10	1.00								
	5	0.70								
Genotype 2	737	2.87	3.05	0.12 [3.86]	0.19	-0.09	2.87	0.12 [4.05]	0	-0.05
	74	1.87	2.13	0.10 [4.50]	0.26	-0.02	1.91	0.13 [6.77]	0.04	-0.01
	37	1.57	1.90	0.08 [4.31]	0.33	0.05	1.57	0.14 [11.52]	0	-0.05
	18	1.26	1.61	0.16 [10.13]	0.34	0.06	1.42	0.15 [10.29]	0.16	0.11
	11	1.05								
	7	0.87								
	4	0.57								
Genotype 3	585	2.77	2.97	0.06 [2.16]	0.21	-0.04	2.97	0.10 [3.68]	0.21	-0.04
	59	1.76	2.02	0.08 [4.07]	0.26	0.01	2.02	0.14 [8.00]	0.26	0.01
	29	1.46	1.75	0.14 [8.04]	0.29	0.04	1.75	0.14 [9.30]	0.29	0.04
	15	1.16								
	9	0.94								
	6	0.77								
	3	0.46								
Genotype 4	558	2.75	2.74	0.07 [2.64]	0	-0.07	2.52	0.09 [3.71]	-0.23	-0.08
	56	1.75	1.83	0.11 [6.08]	0.08	0.01	1.61	0.15 [9.56]	-0.13	0.01
	28	1.45	1.57	0.16 [10.16]	0.13	0.06	1.38	0.12 [8.41]	-0.06	0.08
	14	1.14								
	8	0.92								
	6	0.75								
	3	0.45								

[a] Calculated for all measurements from samples with nominal concentrations above LoD/LLoQ (i.e., above 1.18 $\log_{10}$ IU)

[b] for GT2, 3, and 4, the values for the expected concentrations of each panel member were assigned using a linearization method on the mean observed titers fro

[c] Accuracy = Observed Mean ($\log_{10}$ IU/mL) – Nominal Concentration ($\log_{10}$ IU/mL)

[d] Linearity = Observed Mean ($\log_{10}$ IU/mL) – Linearized ($\log_{10}$ IU/mL)

SD = standard deviation; CV = coefficient of variation

Table S3: Limit of Detection (LoD) and Observed HCV RNA concentration for genotypes 1, 2, 3, and 4 by CAP/CTM v.2

			Observed HCV RNA Concentration (log_{10} IU/mL)[a]				
	Nominal Titer (IU/mL)[b]	Nominal Titer (log_{10} IU/mL)	% Detection of HCV (# of n=40 replicates)	Mean	SD [CV%]	Accuracy[c]	Linearity[d]
Genotype 1	1000	3.00	100 (40)	3.13	0.06 [1.90]	0.13	0.04
	100	2.00	100 (40)	2.10	0.13 [6.23]	0.1	0.02
	50	1.70	100 (40)	1.69	0.19 [11.36]	-0.01	-0.09
	25	1.40	100 (40)	1.47	0.13 [9.19]	0.07	-0.02
	15	1.18	92.5 (37)	1.32	0.12 [8.90]	0.14	0.06
	10	1.00	87.5 (35)				
	5	0.70	50 (20)				
	**LoD by Probit [95% CI]**		**14.4 [12.0, 19.0]**				
Genotype 2	737	2.87	100 (40)	3.15	0.06 [2.05]	0.28	0.03
	74	1.87	100 (40)	2.11	0.14 [6.50]	0.25	-0.01
	37	1.57	100 (40)	1.81	0.20 [10.85]	0.24	-0.01
	18	1.26	100 (40)	1.51	0.20 [13.32]	0.24	-0.01
	11	1.05	100 (40)				
	7	0.87	92.5 (37)				
	4	0.57	62.5 (25)				
	**LoD by Probit [95% CI]**		**7.6 [6.2, 11.5]**				
Genotype 3	585	2.77	100 (40)	3.04	0.05 [1.75]	0.27	0.01
	59	1.76	100 (40)	2.05	0.11 [5.52]	0.28	0.03
	29	1.46	100 (40)	1.68	0.16 [9.57]	0.22	-0.04
	15	1.16	100 (40)				
	9	0.94	97.5 (39)				
	6	0.77	100 (40)				
	3	0.46	80 (32)				
	**LoD by Probit [95% CI]**		**5.0 [3.8, 9.0]**				
Genotype 4	558	2.75	100 (40)	2.95	0.06 [2.02]	0.21	0.05
	56	1.75	100 (40)	1.90	0.23 [11.93]	0.15	-0.01
	28	1.45	100 (40)	1.55	0.18 [11.31]	0.11	-0.05
	14	1.14	90 (36)				
	8	0.92	70 (28)				
	6	0.75	57.5 (23)				
	3	0.45	52.5 (21)				
	**LoD by Probit [95% CI]**		**19.5 [14.5, 32.6]**				

[a] Calculated for all measurements from samples with nominal concentrations above LoD/LLoQ (i.e., above 1.18 log_{10} IU)

[b] for GT2, 3, and 4, the values for the expected concentrations of each panel member were assigned using a linearization method on the mean observed titers from Abbott m2000 HCV, and rounded to the nearest IU

[c] Accuracy = Observed Mean (log_{10} IU/mL) – Nominal Concentration (log_{10} IU/mL)

[d] Linearity = Observed Mean (log_{10} IU/mL) – Linearized (log_{10} IU/mL)

SD = standard deviation; CV = coefficient of variation

Table S4: Limit of Detection (LoD) and Observed HCV RNA concentration for genotype 1 by HPS/CTM v2

		Observed HCV RNA Concentration (log_{10} IU/mL) [a]				
**Nominal Titer (IU/mL)**	**Nominal Titer (log_{10} IU/mL)**	**% Detection of HCV (# of n=40 replicates)**	**Mean**	**SD [CV%]**	**Accuracy [b]**	**Linearity [c]**
1000	3.00	100 (40)	3.03	0.06 [2.12]	0.03	-0.06
100	2.00	100 (40)	2.10	0.10 [4.97]	0.1	0.01
50	1.70	100 (40)	1.79	0.11 [5.94]	0.09	0
25	1.40	100 (40)	1.54	0.12 [7.54]	0.15	0.05
15	1.18	100 (40)				
10	1.00	100 (40)				
5	0.70	90 (36)				
**LoD by Probit [95% CI]**		**6.81 [0.26, 14.59]**				

[a] Calculated for all measurements from samples with nominal concentrations above LoD/LLoQ (i.e., above 1.18 log_{10} IU)

[b] Accuracy = Observed Mean (log_{10} IU/mL) – Nominal Concentration (log_{10} IU/mL)

[c] Linearity = Observed Mean (log_{10} IU/mL) – Linearized (log_{10} IU/mL)

SD = standard deviation; CV = coefficient of variation

Table S5. Distribution of sample results across cobas 6800/8800 HCV versus CAP/CTM HCV v2 (log_{10} IU/mL)

cobas 6800/8800 HCV	CAP/CTM v2				
	Target not detected	< 15 IU/mL	15 to 1E+08 IU/mL	> 1E+08 IU/mL	Total
**Target not detected**	45	1	0	0	46
**< 15 IU/mL**	2	2	1	0	5
**15 to 1E+08 IU/mL**	1	8	185	0	194
**> 1E+08 IU/mL**	0	0	0	0	0
**Total**	48	11	186	0	245

Note: The lower limit of quantitation (LLOQ) of both cobas 6800/8800 HCV and CAP/CTM v2 is 15 IU/mL. The common linear range of both assays is 15 to 1E+08 IU/mL.

CAP/CTM v2 = COBAS® AmpliPrep/COBAS® TaqMan® HCV Quantitative Test, version 2.0; HCV= hepatitis C virus

Table S6. Distribution of sample results across cobas 6800/8800 HCV versus HPS/CTM v2 (log_{10} IU/mL)

cobas 6800/8800 HCV	HPS/CTM v2				
	**Target not detected**	**< 25 IU/mL**	**25 to 1E+08 IU/mL**	**> 1E+08 IU/mL**	**Total**
**Target not detected**	42	4	0	0	46
**< 25 IU/mL**	1	6	1	0	8
**25 to 1E+08 IU/mL**	1	12	177	0	190
**> 1E+08 IU/mL**	0	0	0	0	0
**Total**	44	22	178	0	244

Note: The lower limit of quantitation (LLOQ) of cobas 6800/8800 HCV is 15 IU/mL and for HPS/CTM v2 it is 25 IU/mL. The common linear range of both assays is 25 to 1E+08 IU/mL.

HCV= hepatitis C virus; HPS/CTM v2 = COBAS® TaqMan® HCV Test, version 2 for use with the High Pure System

Table S7. Distribution of sample results across cobas 6800/8800 HCV versus cobas 4800 HCV (log_{10} IU/mL)

cobas 4800 HCV	cobas 6800/8800 HCV				
	**Target not detected**	**< 15 IU/mL**	**15 to 1E+08 IU/mL**	**> 1E+08 IU/mL**	**Total**
**Target not detected**	38	1	2	0	41
**< 15 IU/mL**	3	0	6	0	9
**15 to 1E+08 IU/mL**	1	2	174	0	177
**> 1E+08 IU/mL**	0	0	0	0	0
**Total**	42	3	182	0	227

Note: The lower limit of quantitation (LLOQ) for both cobas 4800 HCV and cobas 6800/8800 HCV is 15 IU/mL. The common linear range of both assays is 15 to 1E+08 IU/mL.

HCV = hepatitis C virus

Table S8. Distribution of sample results across cobas 4800 HCV versus CAP/CTM v2 (log_{10} IU/mL)

cobas 4800 HCV	CAP/CTM v2				
	Target not detected	< 15 IU/mL	15 to 1E+08 IU/mL	> 1E+08 IU/mL	Total
**Target not detected**	41	1	0	0	42
**< 15 IU/mL**	3	2	4	0	9
**15 to 1E+08 IU/mL**	1	6	170	0	177
**> 1E+08 IU/mL**	0	0	0	0	0
**Total**	45	9	174	0	228

Note: The lower limit of quantitation (LLOQ) for both cobas 4800 HCV and CAP/CTM v2 is 15 IU/mL. The common linear range of both assays is15 to 1E+08 IU/mL.

CAP/CTM v2 = COBAS[®] AmpliPrep/COBAS[®] TaqMan[®] HCV Quantitative Test, version 2.0; HCV= hepatitis C virus

Table S9. Distribution of sample results across cobas 4800 HCV versus HCV HPS v2 (log_{10} IU/mL)

cobas 4800 HCV	HPS/CTMv2				
	Target Not Detected	< 25 IU/mL	25 to 1E+08 IU/mL	> 1E+08 IU/mL	Total
**Target Not Detected**	39	3	0	0	42
**< 25 IU/mL**	1	14	4	0	19
**25 to 1E+08 IU/mL**	0	4	162	0	166
**> 1E+08 IU/mL**	0	0	0	0	0
**Total**	40	21	166	0	227

Note: The lower limit of quantitation (LLOQ) cobas 4800 HCV is 15 IU/mL and for HCV HPS v2 it is 25 IU/mL. The common linear range of both assays is 25 to 1E+08 IU/mL.

HCV= hepatitis C virus; HPS/CTM v2 = COBAS® TaqMan® HCV Test, version 2 for use with the High Pure System.

Table S10. Distribution of sample results across HPS/CTM v2 versus CAP/CTM v2 (log_{10} IU/mL)

HPS/CTM v2	CAP/CTM v2				
	Target Not Detected	< 25 IU/mL	25 to 1E+08 IU/mL	> 1E+08 IU/mL	Total
**Target Not Detected**	44	0	0	0	44
**< 25 IU/mL**	5	12	6	0	23
**25 to 1E+08 IU/mL**	0	6	172	0	178
**> 1E+08 IU/mL**	0	0	0	0	0
**Total**	49	18	178	0	245

Note: The lower limit of quantitation (LLOQ) of HPS/CTM v2 is 25 IU/mL and for CAP/CTM v2 it is 15 IU/mL. Hence, the common linear range of both assays is 25 to 1E+08 IU/mL.

CAP/CTM v2 = COBAS® AmpliPrep/COBAS® TaqMan® HCV Quantitative Test, version 2.0; HCV = hepatitis C virus; HPS/CTM v2 = COBAS® TaqMan® HCV Test, version 2 for use with the High Pure System.

Figure S1. Limit of detection for cobas 6800/8800 HCV for genotype 1

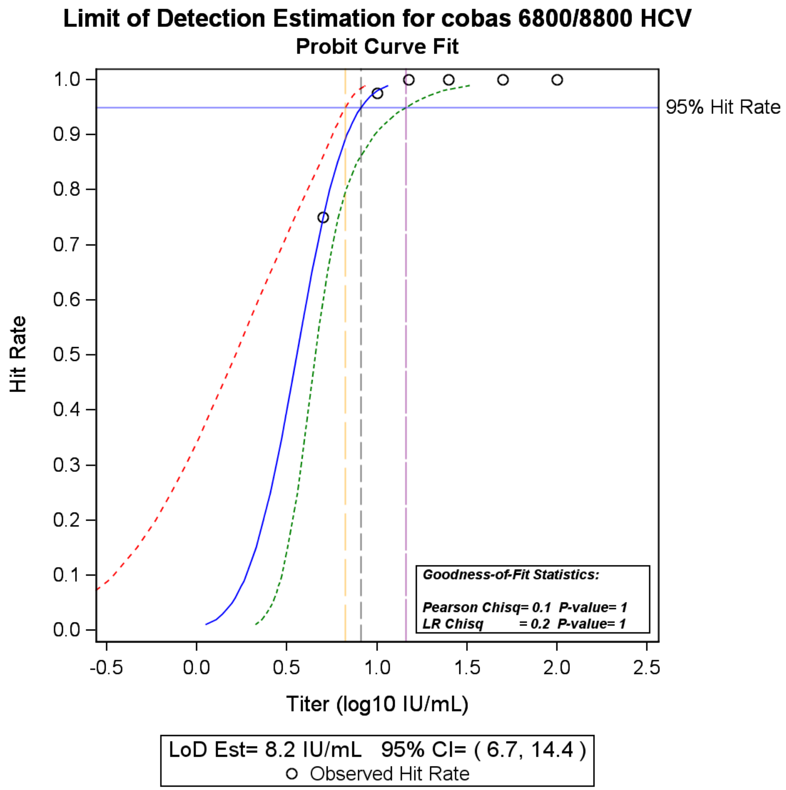

CI = confidence interval; HCV= hepatitis C virus; LOD = limit of detection

Figure S2. Limit of detection for cobas 4800 HCV for genotype 1

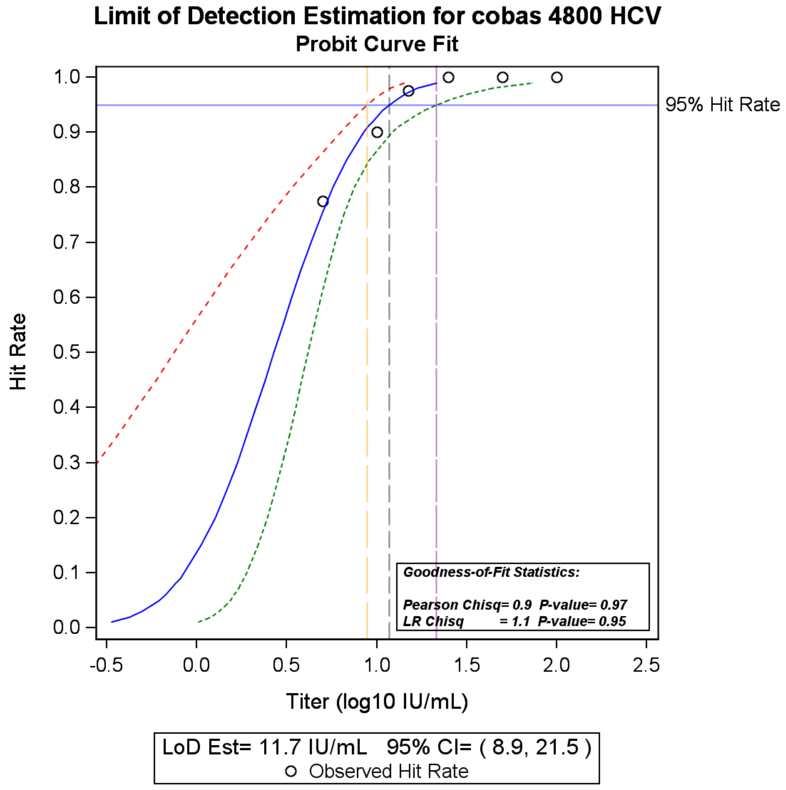

CI = confidence interval; HCV= hepatitis C virus; LOD = limit of detection

Figure S3. Limit of detection estimation for CAP/CTM v2 for genotype 1

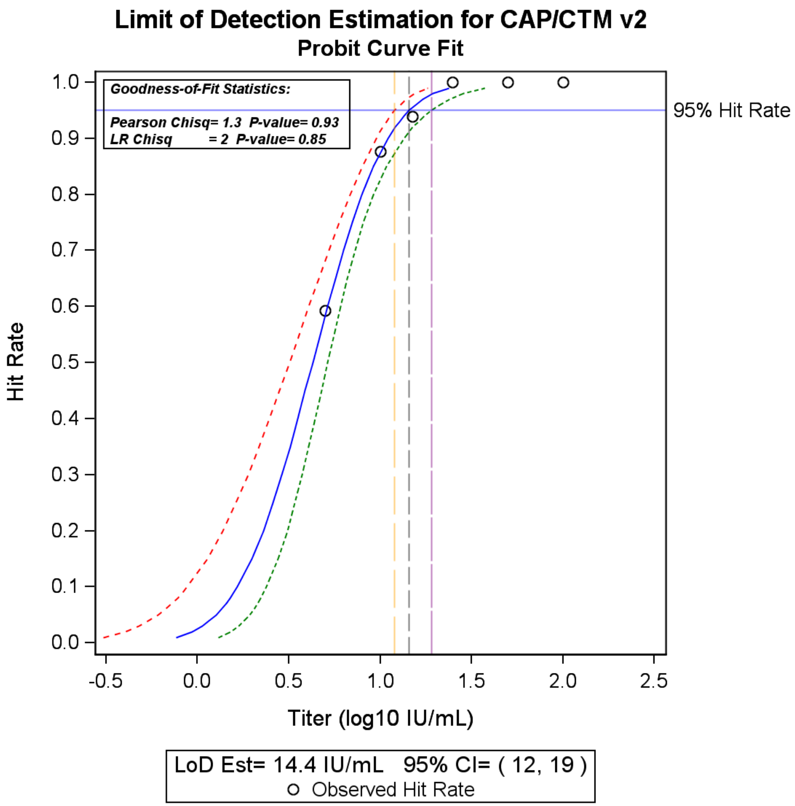

CAP/CTMv2 = COBAS® AmpliPrep/COBAS® TaqMan® HCV Quantitative Test, version 2.0; CI = confidence interval; HCV= hepatitis C virus; LOD = limit of detection

Figure S4. Limit of detection estimation for HPS/CTM v2 for genotype 1

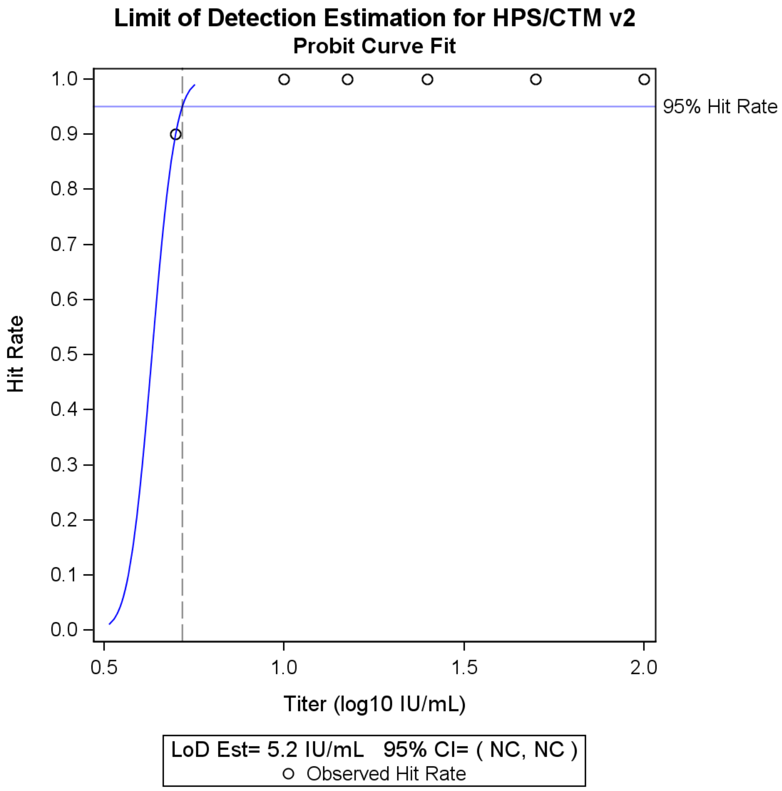

CI = confidence interval; HCV= hepatitis C virus; HPS/CTMv2 = COBAS® TaqMan® HCV Test, version 2 for use with the High Pure System; LOD = limit of detection

Figure S5a. Deming regression plot for cobas 4800 HCV versus CAP/CTM v2 (n=170)

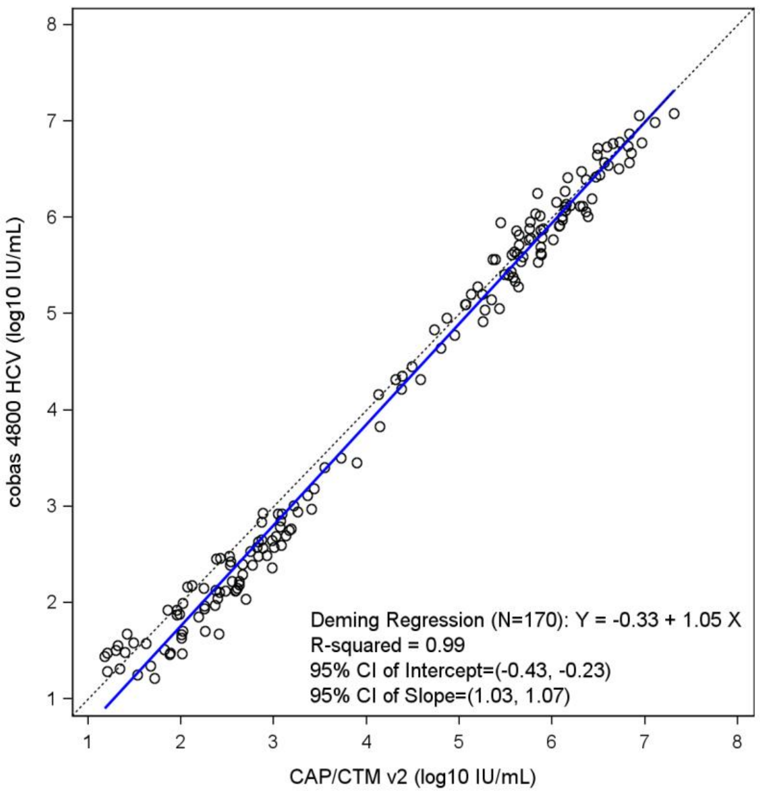

Figure S5b. Bland Altman plot for cobas 4800 HCV versus CAP/CTM v2 (n=170)

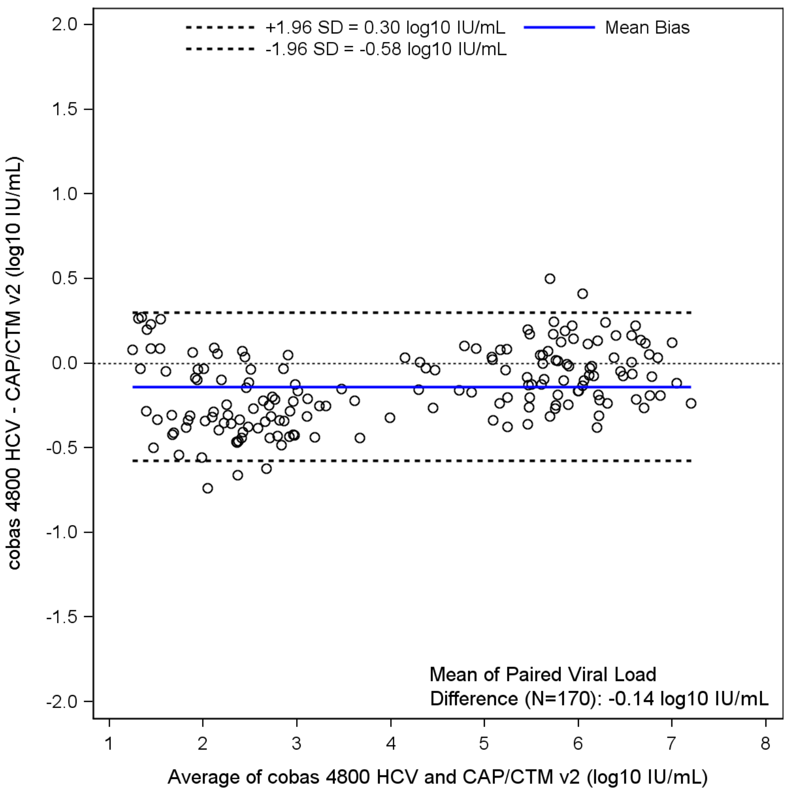

CAP/CTMv2 = COBAS® AmpliPrep/COBAS® TaqMan® HCV Quantitative Test, version 2.0; CI = confidence interval; HCV= hepatitis C virus

Figure S6a. Deming regression plot for cobas 4800 HCV versus HPS/CTM v2 (n=162)

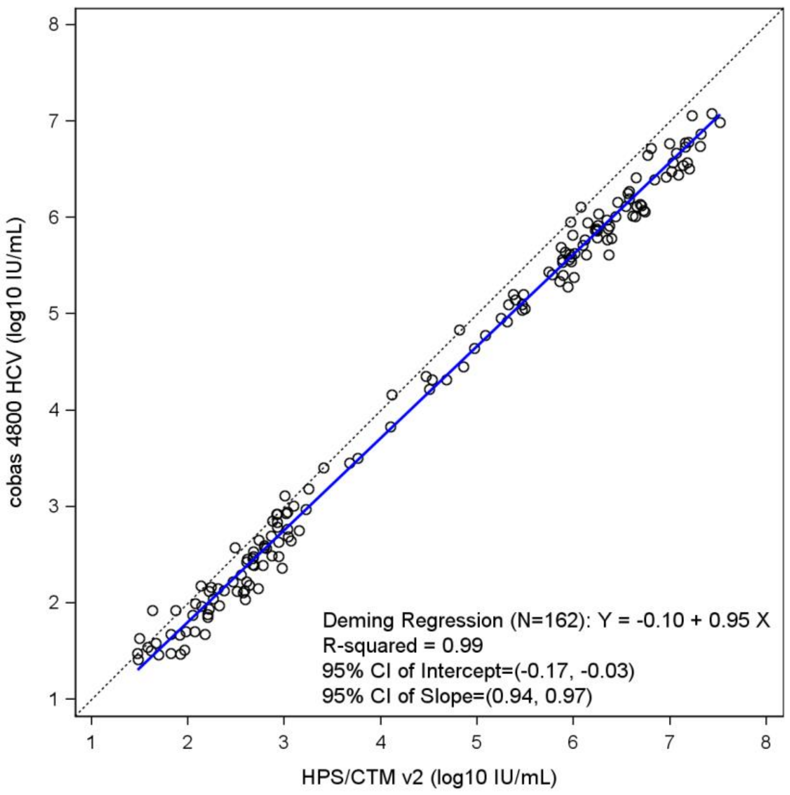

Figure S6b. Bland Altman plot for cobas 4800 HCV versus HPS/CTM v2 (n=162)

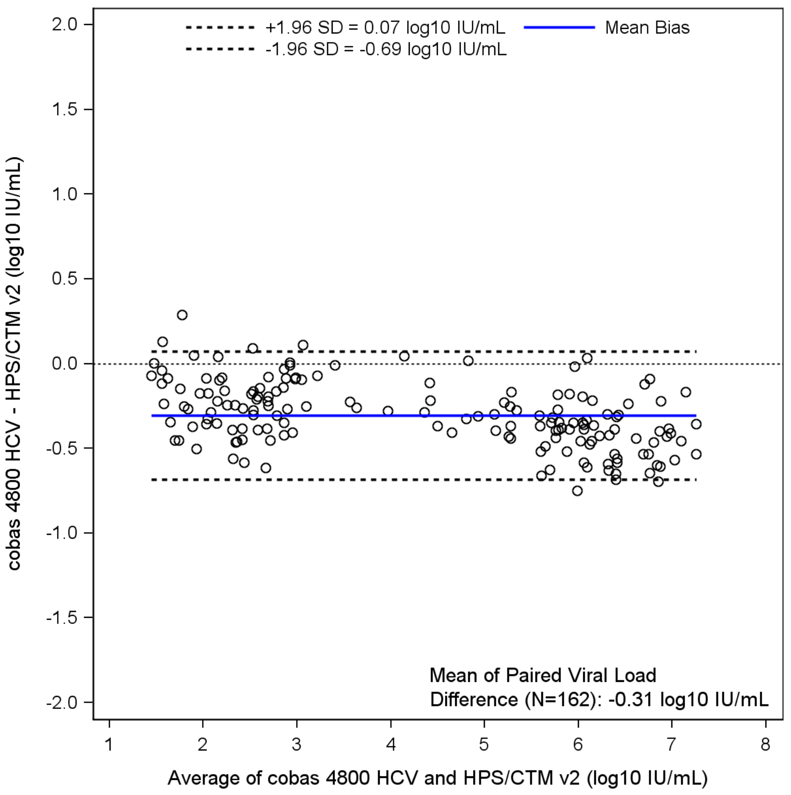

CI = confidence interval; HCV= hepatitis C virus; HPS/CTMv2 = COBAS® TaqMan® HCV Test, version 2 for use with the High Pure System

Figure S7a. Deming regression plot for HPS/CTM v2 versus CAP/CTM v2 (n=172)

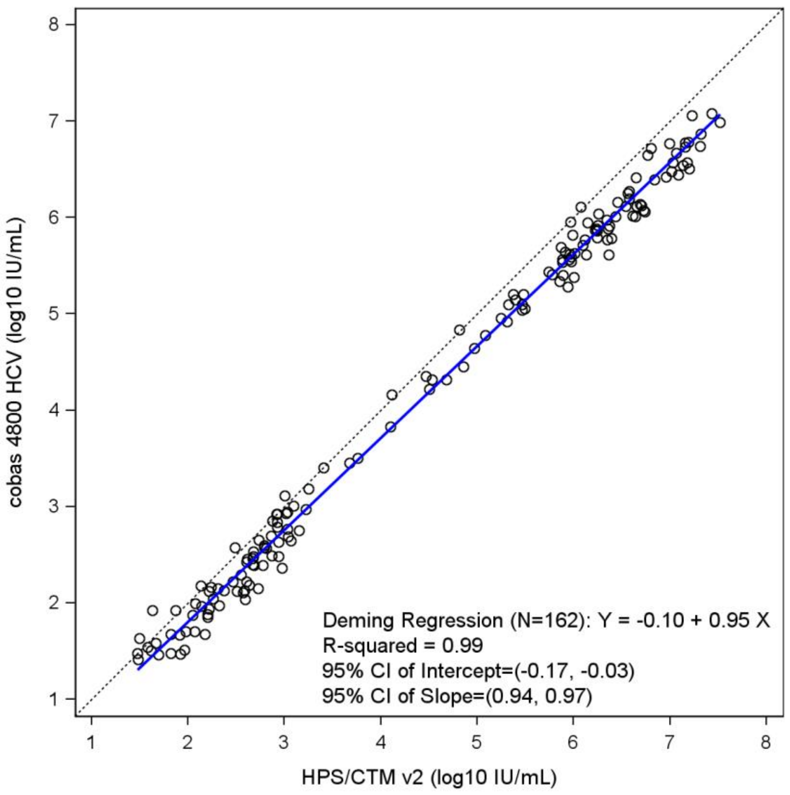

Figure S7b. Bland Altman plot for HPS/CTM v2 versus CAP/CTM v2 (n=172)

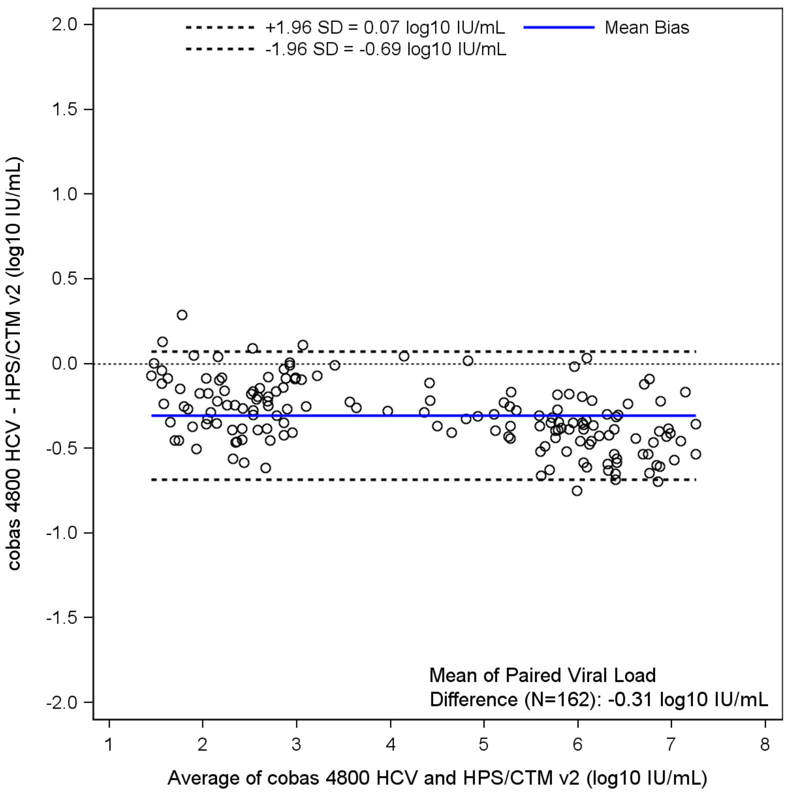

CAP/CTMv2 = COBAS® AmpliPrep/COBAS® TaqMan® HCV Quantitative Test, version 2.0; CI = confidence interval; HCV= hepatitis C virus; HPS/CTMv2 = COBAS® TaqMan® HCV Test, version 2 for use with the High Pure System
